# Concurrent chemoradiotherapy combined with enteral nutrition support: a radical treatment strategy for esophageal squamous cell carcinoma patients with malignant fistulae

**DOI:** 10.1186/s40880-016-0171-6

**Published:** 2017-01-11

**Authors:** Li Ma, Guang-Yu Luo, Yu-Feng Ren, Bo Qiu, Hong Yang, Chun-Xia Xie, Song-Ran Liu, Shi-Liang Liu, Zhao-Lin Chen, Qun Li, Jian-Hua Fu, Meng-Zhong Liu, Yong-Hong Hu, Wen-Feng Ye, Hui Liu

**Affiliations:** 1 Sun Yat-sen University Cancer Center, State Key Laboratory of Oncology in South China, Collaborative Innovation Center for Cancer Medicine, Guangzhou, 510060 Guangdong P. R. China; 2 Guangdong Esophageal Cancer Research Institute, Guangzhou, 510060 Guangdong P. R. China; 3 Department of Radiation Oncology, Sun Yat-sen University Cancer Center, 651 Dongfeng Road East, Guangzhou, 510060 Guangdong P. R. China; 4 Department of Endoscopy, Sun Yat-sen University Cancer Center, Guangzhou, 510060 Guangdong P. R. China; 5 Department of Radiation Oncology, The First Affiliated Hospital of Sun Yat-sen University, Guangzhou, 510080 Guangdong P. R. China; 6 Department of Thoracic Surgery, Sun Yat-sen University Cancer Center, Guangzhou, 510060 Guangdong P. R. China; 7 Department of Clinical Nutrition, Sun Yat-sen University Cancer Center, 651 Dongfeng Road East, Guangzhou, 510060 Guangdong P. R. China

**Keywords:** Esophageal squamous cell carcinoma, Malignant fistula, Radiotherapy, Concurrent chemotherapy, Enteral nutrition support

## Abstract

**Background:**

Concurrent chemoradiotherapy (CCRT) significantly increases the survival rate of esophageal squamous cell carcinoma (ESCC) patients with malignant fistulae. Recent clinical evidence has shown the benefits of enteral nutrition for malnourished cancer patients. In this study, we aimed to validate that, with the support of enteral nutrition, ESCC patients who develop malignant fistulae might be able to complete CCRT and achieve long-term survival.

**Methods:**

We reviewed the medical records of 652 patients with ESCC who received definitive CCRT at Sun Yat-sen University Cancer Center between January 2010 and December 2012. Treatment outcome and toxicity were retrospectively evaluated in 40 ESCC patients with malignant fistulae. All the 40 patients were treated with CCRT and evaluated by clinical nutritionists using nutrition risk screening (NRS) before, during, and after treatment. Twenty-two patients received a nasogastric tube, and 18 underwent percutaneous endoscopic gastrostomy feeding. The median energy intake was 2166 kcal/day. Treatment response was evaluated at 3 months after the completion of CCRT.

**Results:**

With a median follow-up of 18 months (range, 3–39 months), patients’ 1-year overall survival (OS) rate was 62.5%, and the estimated OS time was 25.5 months. Univariate analysis showed that the NRS score (*P* = 0.003), increase in NRS score (*P* = 0.024), fistula closure (*P* = 0.011), and response to treatment (*P* < 0.001) were significantly associated with OS. Multivariate analysis showed that tumor response (*P* = 0.044) and increase in NRS score (*P* = 0.044) were independent predictors of OS. Grade 3 vomiting was observed in 8 patients (20.0%), grade 3 neutropenia was observed in 11 patients (27.5%), and grade 3 cough was observed in 13 patients (32.5%); 2 patients (5.0%) died of massive bleeding during treatment.

**Conclusions:**

CCRT combined with enteral nutrition support is effective for ESCC patients with malignant fistulae. Patients have an increased potential to be cured, especially those who experience complete response and have an increase in NRS score. Careful observation and nutrition support are required for patients with advanced T-category ESCC who undergo CCRT.

## Background

Esophageal cancer is among 10 most common causes of cancer-related death in China [1]. Malignant fistulae between the esophagus and respiratory tract (ER fistula) or between the esophagus and mediastinum (EM fistula) are serious complications for patients with esophageal carcinoma [2]. Bronchopneumonia, sepsis, and massive bleeding are the most common terminal events observed in patients with a malignant fistula. Many patients with malignant fistulae eventually die, with a short median survival ranging from 1 to 6 weeks [3, 4]. Historically, the presence of ER fistula or EM fistula has been considered a relative contraindication for radiotherapy and chemotherapy. However, a large retrospective analysis showed that, for esophageal squamous cell carcinoma (ESCC) patients with malignant fistulae, radiotherapy significantly extended overall survival (OS) compared with supportive care [4]. Although most concurrent chemoradiotherapy (CCRT) trials for ESCC patients have excluded primary tumors with fistulae [5, 6], some studies reported that, for patients with locally advanced esophageal carcinoma, significant improvement in local control and OS can be achieved with CCRT compared with radiotherapy alone [7–9]. Koike et al. [10] reported that CCRT with protracted cisplatin and 5-fluorouracil (5-FU) infusion for patients with T4 esophageal carcinoma and malignant fistulae could lead to a 2-year survival rate of 22%.

Malnutrition is a common comorbidity in esophageal carcinoma patients, affecting up to 80% of patients at the time of diagnosis; for esophageal carcinoma patients with malignant fistulae, nutritional status is even worse [11]. Although CCRT has become a standard treatment strategy for esophageal carcinoma, this regimen is associated with several toxicities, such as bone marrow suppression, esophagitis, mucositis, nausea, and vomiting [7–10]. Recent clinical evidence has shown the benefits of enteral nutrition in malnourished cancer patients: it can maintain quality of life and improve nutritional status by ensuring adequate nutrient intake [12–14]. Therefore, with the support of enteral nutrition, ESCC patients who develop malignant fistulae might be able to complete CCRT and achieve long-term survival.

Building on our previous work that made the promising effects of CCRT on esophageal carcinoma [15, 16], we treated patients who had advanced esophageal carcinoma and ER or EM fistulae with aggressive CCRT combined with enteral nutrition support. Here, we reviewed and analyzed the clinical results of this treatment strategy for esophageal carcinoma patients who developed malignant fistulae before or during treatment.

## Patients and methods

### Patient and clinical data

We reviewed the medical records of 652 patients with ESCC who received definitive CCRT at Sun Yat-sen University Cancer Center, in Guangzhou, Guangdong, China between January 2010 and December 2012. Among 652 patients, 73 ESCC patients were identified to have fistulae. The patients were diagnosed according to the American Joint Committee on Cancer tumor, node, and metastasis (TNM) classification (7th edition). All the patients who met the following inclusion criteria were included in this study: (1) confirmed thoracic ESCC by pathologic analysis; (2) no previous cancer treatments and no distant metastases; (3) Eastern Cooperative Oncology Group (ECOG) performance status ≤2; (4) a complete evaluation, including physical examination, computed tomography (CT) scanning of the chest and abdomen, an upper gastrointestinal barium meal exam, and endoscopic ultrasound of the esophagus; and (5) meglumine diatrizoate mucilage (MDC) leakage with/without endoscopy for fistulae assessment.

Clinical data collected from each patient included ECOG performance status, a nutrition assessment, age, sex, primary esophageal tumor location, clinical stage and T category of primary tumor, radiation dose, CCRT regimen, and tumor response to CCRT.

### Enteral nutrition support and assessment

Before, during, and after CCRT, all patients were evaluated by clinical nutritionists using nutrition risk screening (NRS). Patients were evaluated in terms of undernutrition and disease severity, according to whether they are absent, mild, moderate, or severe, making a total score of 0–6; patients with a total score of ≥3 were classified as nutritionally at-risk. Undernutrition was estimated using three variables used in most screening tools: body mass index (BMI), percent of recent weight loss, and change in food intake. Diseases like hip fracture, chronic diseases, and tumor were scored 1; major abdominal surgery, stroke, diabetes, and hematologic malignancy were scored 2; head injury and bone marrow transplantation were scored 3 [17]. Increased NRS score indicated improved nutritional status of patients than before. When diagnosed with a malignant fistula, patients were administered enteral nutrition support. The patients either received nasal feeding or underwent a percutaneous endoscopic gastrostomy (PEG), and therefore dietary intake could be adjusted and to achieve energy balance and minimize weight loss based on patient weight that was continuously monitored.

Intacted protein enteral nutrition powder formula (Danone; Paris, France) was used for nasal feeding (each 500-mL bottle provides 20 g of protein and 500 kcal of energy). Oral and/or enteral energy-rich and protein-rich supplements were added when needed. At all measurement points, the PEG stoma site was observed and care advice was given when needed. Nutritional supplements were administered until 4–8 weeks after fistula closure.

All laboratory test values, including hemoglobin level and serum albumin level, were determined in the clinical laboratories of Sun Yat-sen University Cancer Center.

### Radiotherapy and concurrent chemotherapy

During radiotherapy, the techniques used for patient immobilization, simulation, and treatment planning were performed according to a standard protocol in the Department of Radiotherapy at Sun Yat-sen University Cancer Center for esophageal carcinoma patients receiving three-dimensional conventional radiotherapy (3D-CRT) [18]. With the patient in the supine position, a cradle for immobilization was made with a vacuum. Individual patients were scanned from the atlas (C1) to the second lumbar vertebra (L2) level to cover the entire neck, lung, esophagus, and celiac lymph node regions. CT scans were performed with 0.5-cm thickness slices. Briefly, the gross tumor volume (GTV-esophagus) consisted of lesions diagnosed by biopsy or subsequent CT scans; tumor regions described on endoscopy but not observed on CT were also included in the GTV-esophagus. The criteria for GTV of positive lymph nodes (GTV-ln) based on CT scans were as follows: short axis size ≥10 mm, a lymph node with an infiltrative margin, or central necrosis. Two clinical target volumes (CTVs) for the patients were defined: CTV1 comprised GTV-ln and 2 cm proximal and distal to the GTV-esophagus; CTV2 comprised the supraclavicular and mediastinal lymph nodes, GTV-ln, and 4 cm proximal and distal to the GTV-esophagus. PTV1 was defined as a 5-mm margin added to CTV1; PTV2 was defined as a 5-mm margin added to CTV2 [17]. All patients had a 3D-CRT treatment plan that was calculated by the Pinnacle treatment planning system, and they were treated with a 6-MV linear accelerator (MIMiC; Nomos Corp., Sewickly, PA, USA). The median dose was 60 Gy for GTV (range, 46–68 Gy), 55 Gy for PTV1 (range, 40–68 Gy), and 46 Gy for PTV2 (range, 40–54 Gy). Dose constraints for critical organs were as follows: the maximum spinal cord dose <46 Gy, mean lung dose <17 Gy, and the lung volumes irradiated above 20 Gy (V20) <30%.

Two regimens of chemotherapy were used in the study: (1) concurrent chemotherapy consisted of cisplatin (20 mg/m^2^ per day) and 5-FU (500 mg/m^2^ per day), every 3 weeks; (2) docetaxel-based regimens consisted of docetaxel (60 mg/m^2^ per day) and cisplatin (60 mg/m^2^ per day), every 3 weeks; or concurrent chemotherapy comprising cisplatin (25 mg/m^2^ per day) and docetaxel (25 mg/m^2^ per day), weekly [15, 16].

### Follow-up and treatment response assessment

The beginning of the follow-up period was defined as the last date of CCRT treatment. During the follow-up period, patients underwent a chest CT scan every 3 months, an upper digestive tract endoscopy and an abdominal ultrasonography every 6 months for 2 years after CCRT, and a subsequent chest CT scan, an endoscopy, and an abdominal ultrasonography every 6 months thereafter. Bone scans were performed when patients were suspected to have bone metastases. The rates and time to treatment response or distant metastasis, duration of OS and local relapse were recorded.

MDC leakage evaluation and/or endoscopy were performed every 2–3 weeks from the diagnosis of malignant fistulae until 4 weeks after fistula closure.

Tumor response evaluations were performed 1–3 months after CCRT according to response evaluation criteria in solid tumors (RECIST) definitions. For the primary tumors, the responses include complete response (CR), partial response (PR), progressive disease (PD), and stable disease (SD) [15, 19]. Multiple failures comprised both local and distant failures after CCRT. Acute toxicity was graded using the National Cancer Institute Common Toxicity Criteria (version 4.0).

### Statistical analysis

The study endpoint was OS, which was calculated as the time from the last date of radiotherapy to the date of death from any cause or to the date of the last visit before September 30, 2013. Continuous variables, such as age, hemoglobin level, serum albumin level, and radiation dose, were normalized as the sample median and then analyzed as nominal categorical variables. Each variable was assessed first in univariate analysis, and variables that reached a *P* value of less than 0.05 were further evaluated in multivariate analysis. Survival curves were plotted using the Kaplan–Meier method. We fitted the proportional hazards model using Cox regression. After testing for variable interactions, a forward stepwise elimination procedure was used to determine the best-fitting model. In the multivariate analysis*, P* values less than 0.05 were considered statistically significant. All statistical analyses were performed using SPSS 19.0 software (IBM, Chicago, IL, USA).

### Ethics statement

Our thoracic multi-disciplinary team discussed the treatment of all patients. Written informed consent was not obtained; instead, all clinical records were anonymized and de-identified prior to analysis. The entire study was approved by the Ethics Committee of Sun Yat-sen University Cancer Center.

## Results

### Patient characteristics

Forty ESCC patients (37 men and 3 women) were pathologically diagnosed with malignant fistulae and were finally included in this study. Patient characteristics are detailed in Table 1. Most primary lesions (26/40, 65.0%) were located in the middle thoracic esophagus. Twenty-two patients had stages III and IV disease, and 18 patients had stages I and II disease; 5 had T1-2 lesion, 21 had T3 lesion, and 14 had T4 lesion. The NRS scores of 22 patients were 3–4 (moderate to severe impaired nutritional status) before treatment, and 16 patients experienced an increase in NRS score with nutrition support during CCRT. All patients received concurrent chemotherapy; most (33/40, 82.5%) received a docetaxel-based regimen. The median radiation dose was 60 Gy (range, 46–68 Gy); 12 patients (30.0%) received a lower dose (46–58 Gy). Fistula closure was observed in 32 patients (80.0%). Twelve patients (30.0%) had a CR, and 20 (50.0%) had a PR.

**Table 1:** Characteristics of 40 esophageal squamous cell carcinoma patients with malignant fistulae

Characteristic	No. of patients (%)	1-year OS rate^d^ (%)	*P* value
Sex			0.006
Men	37 (92.5)	67.6	
Women	3 (7.5)	0.0	
Age^a^ (years)	58 (41–80)		
ECOG performance status			0.267
0–1	11 (27.5)	68.2	
2	29 (72.5)	55.2	
Primary tumor location			0.403
Upper	12 (30.0)	50.0	
Middle	26 (65.0)	65.4	
Lower	2 (5.0)	100.0	
T category of primary tumor^b^			0.392
T1	2 (5.0)	100.0	
T2	3 (7.5)	100.0	
T3	21 (52.5)	57.1	
T4	14 (35.0)	57.1	
Clinical stage of primary tumor^b^			0.526
IIA–IIB	2 (5.0)	100	
IIIA–IIIC	29 (72.5)	62.1	
IV	9 (22.5)	55.5	
NRS score of 3–4^c^			
Before nutrition support	22 (55.0)	–^e^	
After nutrition support	6 (15.0)	–	
Hemoglobin level after CCRT^a^ (g/L)	110 (56–156)	–	
Total energy intake^a^ (kcal/day)	2166 (1956–2213)	–	
Total protein intake^a^ (g/kg per day)	1.53 (1.41–1.76)	–	
Fistula closure	32 (80.0)	–	
Time to fistula closure^a^ (weeks)	5 (2–11)	–	
Fistula site			0.435
Trachea and bronchus	7 (17.5)	57.1	
Mediastinum	33 (82.5)	63.6	
Radiation dose^a^ (Gy)	60 (46–68)		<0.001
<60	14 (35.0)	50.0	
≥60	26 (65.0)	69.2	
Concurrent chemotherapy			0.333
DDP + 5-FU	7 (17.5)	42.9	
Docetaxel-based regimens	33 (82.5)	66.7	
Clinical tumor response after CCRT			<0.001
CR	12 (30.0)	91.7	
PR	20 (50.0)	65.0	
SD	3 (7.5)	33.3	
PD	5 (12.5)	0	

ECOG, Eastern Cooperative Oncology Group; NRS, nutrition risk screening; CCRT, concurrent chemoradiotherapy; DDP, cisplatin; 5-FU, 5-fluorouracil; CR, complete response; PR, partial response; SD, stable disease; PD, progressive disease

^a^These values are presented as median followed by range in parentheses. Other values are presented as the number of patients with the percentage in parentheses

^b^Based on the American Joint Committee on Cancer tumor, node, and metastasis (TNM) classification (7th edition)

^c^The patients with NRS score of 1–2 are not listed in this table

^d^Only the values that were compared between subgroups are listed and analyzed

^e^The data were not applicable

### Treatment outcomes

**Fig. 1:**
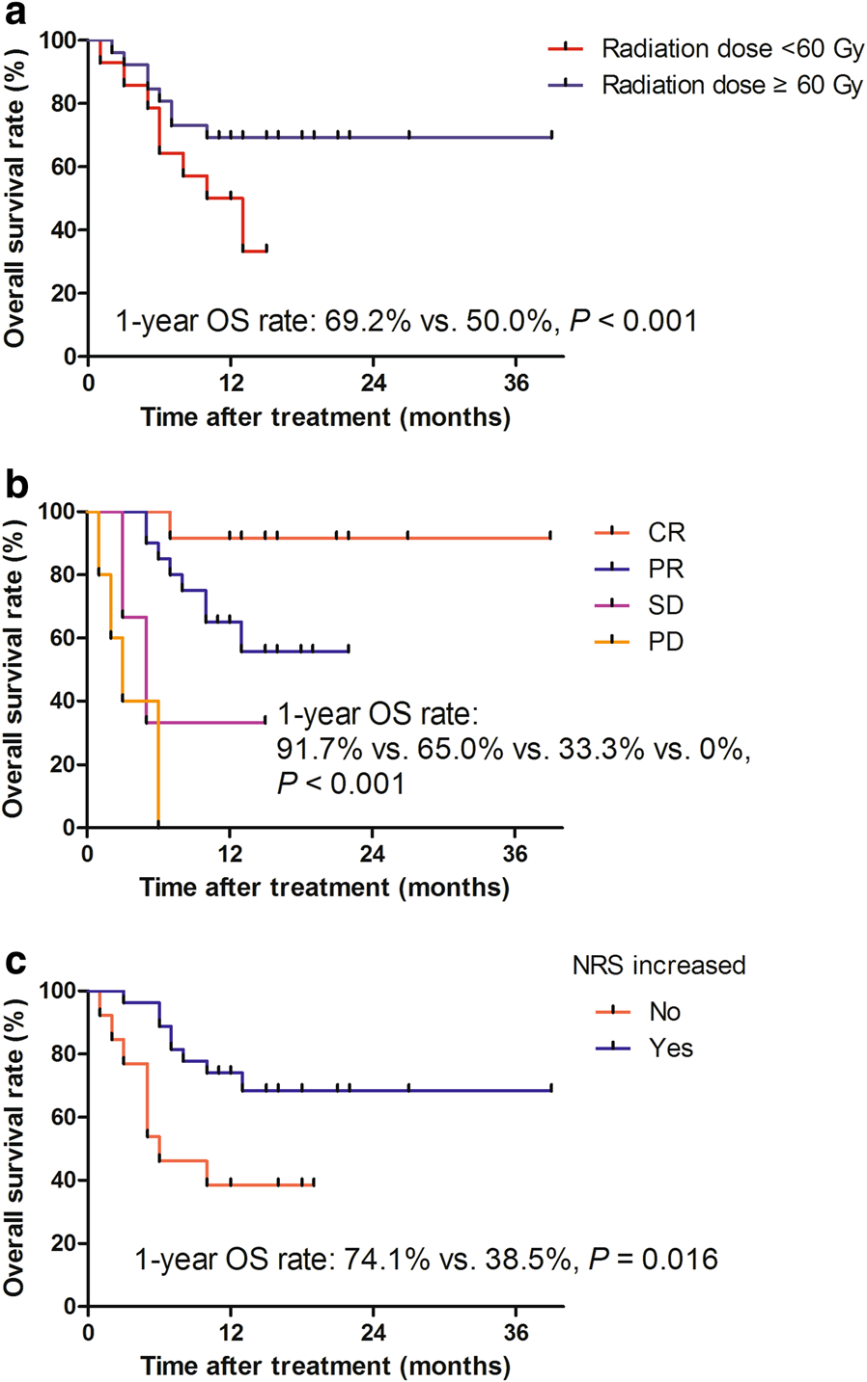
Kaplan–Meier overall survival (OS) curves for esophageal squamous cell carcinoma patients with malignant fistula categorized by radiation dose, response to treatment, and increased nutrition risk screening (NRS) score or not. **a** OS curves for patients receiving different doses of radiation. **b** OS curves for patients with different responses to treatment. **c** OS curves for patients who experienced an increase of NRS score or not. CR, complete response; PR, partial response; SD, stable disease; PD, progressive disease

With a median follow-up of 18 months (range, 3–39 months), the 1-year OS rate of all patients was 62.5%, and the estimated OS was 25.5 months. Univariate analysis showed that, after CCRT completion, NRS score (*P* = 0.003), increase in NRS score (*P* = 0.024), fistula closure (*P* = 0.011), and response to treatment (*P* < 0.001) were significantly associated with OS (Table 2). Clinical factors that were statistically significant (*P* < 0.05) in univariate analysis were further analyzed in a multivariate analysis with the stepwise regression of variables. Only patients who had a tumor response (HR = 3.49, 95% CI 1.48–8.23, *P* = 0.004) and increase in NRS score (HR = 0.23, 95% CI 0.06–0.94, *P* = 0.004) after CCRT were selected by the stepwise addition of factors in the final models. The 1-year OS rates of patients who achieved CR, PR, SD, and PD were 91.7%, 65.0%, 33.3% and 0%, respectively. The 1-year OS rates of patients with an increased and non-increased NRS scores were 74.1% and 38.5%, respectively (Fig. 1a–c). Patients with T4 tumor and fistulae had an 1-year OS rate similar to that of patients with non-T4 tumor and fistulae who received nutrition supported during CCRT (57.1% vs. 69.6%, *P* = 0.198).

**Table 2:** Univariate analysis of prognostic factors of overall survival in 40 esophageal squamous cell carcinoma patients with malignant fistulae

Variable	HR (95% CI)	*P* value
ECOG performance status (0–1 vs. 2)	0.76 (0.26–1.87)	0.567
Primary tumor location (upper vs. middle vs. lower)	0.44 (0.19–1.00)	0.051
T category of primary tumor	1.95 (0.68–5.56)	0.213
(T4 vs. non-T4)		
Clinical stage of primary tumor (stage II vs. stage III vs. stage IV)	1.55 (0.68–3.57)	0.300
NRS score before CCRT (≥3 vs. <3)	1.13 (0.69–1.86)	0.631
NRS score after CCRT (≥3 vs. <3)	5.14 (1.72–15.31)	*0.003*
Increased NRS score after CCRT (yes vs. no)	0.32 (0.12–0.86)	*0.024*
Hemoglobin level after CCRT (>110 vs. ≤110 g/L)	1.56 (0.72–3.24)	0.724
Fistula closure (yes vs. no)	3.78 (1.36–10.61)	*0.011*
Fistula site (tracheobronchus vs. mediastinum)	1.55 (0.50–4.82)	0.447
Radiation dose (≥60 vs. <60 Gy)	0.46 (0.17–1.23)	0.096
Tumor response after CCRT (CR vs. non-CR)	3.53 (2.01–6.18)	<*0.001*

HR, hazard ratio; CI, confidence interval. ECOG, Eastern Cooperative Oncology Group; NRS, nutrition risk screening; CCRT, concurrent chemoradiotherapy; CR, complete response

The italicized *p* values are statistically significant

### Calorie intake and nutritional status

Eighteen patients had malignant fistula before CCRT; 22 patients developed fistula during treatment. In these 22 patients, the median time from the beginning of CCRT to the formation of fistula was 22 days (range, 7–36 days). Patients were given enteral nutrition support when diagnosed with malignant fistula. Nasal feeding was administered to 22 patients; 18 underwent PEG feeding. The median energy intake was 2166 kcal/day; the median protein intake was 1.53 g/kg weight per day (range, 1.41–1.76 g/kg weight per day). Twenty-two patients had an NRS score of 3–4 before CCRT; 6 had an NRS score of 3–4 after treatment. The median time from diagnosis to fistula closure for all 40 patients was 5 weeks.

### Toxicities

The most frequent toxicities observed were vomiting, neutropenia, esophagitis, and cough, with a large majority of toxicity degrees being grade 1 or 2. Grade 3 vomiting, neutropenia, and cough were observed in 8 (20.0%), 11 (27.5%), and 13 patients (32.5%), respectively. Two patients (5.0%) died of massive bleeding during treatment. Two patients (5.0%) developed re-perforation after the initial fistula closure.

## Discussion

Our study demonstrated that CCRT combined with enteral nutrition support offers a cure for malignant fistulae. In our study, 40 patients who underwent CCRT for esophageal carcinoma with a malignant fistula had an estimated OS of 25.2 months, with 62.5% of the patients remaining alive at 1 year after treatment.

CCRT has been used as a primary therapeutic regimen for more and more patients who have unresectable esophageal carcinoma, who decline surgery, or who are deemed medically unfit for surgery. Radiation Therapy Oncology Group 85–01 was the first trial to analyze the efficacy of radiochemotherapy as a definitive treatment, and it demonstrated the superiority of CCRT over radiotherapy alone with regard to 5-year OS rate [20]. Historically, the presence of a malignant esophageal fistula was considered a relative contraindication to CCRT. Ahmed et al. [9] found that malignant ER fistulae could completely be cured in 4 of 5 patients (80%) treated with 5-FU (400–600 mg/m^2^) by protracting continuous infusion and 60-Gy radiotherapy. Muto et al. [8] reported promising CCRT results for patients with malignant esophageal fistulae. Indeed, fistula was closed after CCRT in 17 of 24 patients (71%), with a median OS of 198 days as calculated from the fistula diagnosis. These results suggested that the presence of a malignant fistula was not a contraindication for CCRT, which was the treatment that provided the best chance for survival and palliation of dysphagia.

Most patients were selected for non-surgical therapy because of comorbidity or locally advanced disease. Esophageal perforation may be inevitable when patients with T4 esophageal tumors are treated with conformal radiotherapy. Ishida et al. [5] reported that 6 of 45 patients (13%) with a T4 tumor and/or M1 lymph nodes developed esophageal-bronchial fistulae during CCRT, and CCRT was consequently terminated. Most previous CCRT trials for patients with advanced esophageal carcinoma excluded the patients with fistulae; however, in our study, we found that patients with T4 tumor and fistulae who received nutrition support during CCRT had similar OS to patients with non-T4 tumor and fistulae (1-year OS rate, 57.1% vs. 69.6%, *P* = 0.198).

Furthermore, recent studies showed that the patients who underwent preoperative CCRT followed by surgery and achieved a complete pathologic response to preoperative CCRT had excellent prognosis [21–23]. Although clinical CR after CCRT may not be significantly associated with pathologic CR [24], maximizing the CR rate is likely to increase the proportion of patients with the most favorable outcome, potentially increasing the survival rate of the whole group. In accordance with the published data above, the patients in our study who achieved CR had a 1-year survival rate of 91.7%; in the group of 8 patients who did not achieve CR (SD, *n* = 3; PD, *n* = 5), 7 (87.5%) died within 1 year. These results suggested that even in esophageal carcinoma patients with malignant fistula, achieving clinical CR is a very important prognostic factor of long-term survival. In addition, patients who received a higher radiation dose and more cycles of chemotherapy had a better chance of achieving CR as well as extended OS than patients with lower radiation dose.

For esophageal carcinoma patients, malnutrition might be another important cause of pneumonia and massive bleeding. For patients undergoing chemotherapy and/or radiotherapy, minor malnutrition (weight loss <10%) is also significantly associated with poor prognosis. In a retrospective review of 1555 patients with gastrointestinal malignancies who underwent chemotherapy, Andreyev et al. [25] suggested that weight loss at presentation may be an independent prognostic predictor for developing more severe dose-limiting toxicities (*P* < 0.001), decreased response rate (*P* = 0.006), and shorter OS (*P* < 0.001) for patients with gastric and colorectal neoplasms. For 350 patients with advanced esophageal carcinoma who were treated in 6 consecutive prospective trials, weight loss of more than 5% was a poor prognostic factor (9 vs. 12 months, *P* = 0.006) [26]. According to European Society for Clinical Nutrition and Metabolism guidelines on enteral nutrition for patients who receive external-beam radiotherapy or CCRT, every effort should be made to increase dietary intake and prevent therapy-associated weight loss and radiotherapy interruption [27]. In our study, for most patients, we observed a gain in total mass, involving mainly lean mass, and an increase in weight. Our results also showed that enteral nutrition support increased the NRS score of malnourished patients who received CCRT, and patients who had an increased NRS score had higher 1-year OS rate than those who did not have an increased score (74.1% vs. 38.5%, *P* = 0.016). It is generally thought that radiotherapy interrupts normal wound-healing mechanisms by leading to changes in the vasculature, by affecting fibroblasts, and by varying the levels of regulatory growth factors [28, 29]. Studies of preoperative radiotherapy have shown an increased risk for wound-healing complications compared with postoperative radiotherapy [30]. For this reason, malignant esophageal fistulae were previously regarded as incurable. However, most patients died within 12 months if radiotherapy was terminated [9, 10]. In the present study, the median fistula diameter upon MDC was 4.3 mm, and adequate enteral nutrition support appeared to be sufficient for minor wounds. The median time from diagnosis of fistula to fistula closure was 5 weeks. Two patients developed re-perforation after fistula closure, due to low protein intake after they were released from the hospital. In contrast, 1 patient maintained a good nutrition status (NRS score = 1) even without fistula closure. These results suggest that maintaining good nutritional status is important for fistula closure.

Compared with the results in other published studies [8–10], the higher treatment response and local control rates in our study might be due to the following: (1) the use of the 3D-CRT technique (3D-CRT has a better GTV high-dose coverage compared with two-dimensional conventional radiotherapy, which was used in most previous studies); (2) the higher radiation dose was administered (in the present study, the median dose was 60 Gy, ranging from 46 to 68 Gy; better local control could be achieved with radiation dose greater than 60 Gy in ESCC); (3) all patients received concurrent chemotherapy; and (4) all patients received enteral nutrition support.

This retrospective study has several limitations, such as selection bias, different chemotherapy regimens, small sample size, and short follow-up. However, our results showed that, for ESCC patients, a malignant fistula is not a contraindication for CCRT. When enteral nutrition support is provided together with CCRT, patients can achieve promising improvement and have an increased potential to be cured.

## Conclusions

CCRT combined with enteral nutrition support is an effective treatment regimen for ESCC patients with malignant fistulae, and these patients have an increased potential to be cured, especially those who achieve CR and an increase in NRS score. Careful observation and clinical nutrition support are required for patients with advanced T-category ESCC who underwent CCRT.
